# Evaluation of the similarity of gene expression data estimated with SAGE and Affymetrix GeneChips

**DOI:** 10.1186/1471-2164-6-91

**Published:** 2005-06-14

**Authors:** Fred van Ruissen, Jan M Ruijter, Gerben J Schaaf, Lida Asgharnegad, Danny A Zwijnenburg, Marcel Kool, Frank Baas

**Affiliations:** 1 Department of Neurogenetics, Academic Medical Center, University of Amsterdam, Meibergdreef 9, 1105 AZ Amsterdam, The Netherlands; 2 Department of Anatomy and Embryology, Academic Medical Center, University of Amsterdam, Meibergdreef 9, 1105 AZ Amsterdam, The Netherlands; 3 Department of Human Genetics, Academic Medical Center, University of Amsterdam, Meibergdreef 9, 1105 AZ Amsterdam, The Netherlands

## Abstract

**Background:**

Serial Analysis of Gene Expression (SAGE) and microarrays have found awidespread application, but much ambiguity exists regarding the evaluation of these technologies. Cross-platform utilization of gene expression data from the SAGE and microarray technology could reduce the need for duplicate experiments and facilitate a more extensive exchange of data within the research community. This requires a measure for the correspondence of the different gene expression platforms. To date, a number of cross-platform evaluations (including a few studies using SAGE and Affymetrix GeneChips) have been conducted showing a variable, but overall low, concordance. This study evaluates these overall measures and introduces the between-ratio difference as a concordance measure pergene.

**Results:**

In this study, gene expression measurements of Unigene clusters represented by both Affymetrix GeneChips HG-U133A and SAGE were compared using two independent RNA samples. After matching of the data sets the final comparison contains a small data set of 1094 unique Unigene clusters, which is unbiased with respect to expression level. Different overall correlation approaches, like Up/Down classification, contingency tables and correlation coefficients were used to compare both platforms. In addition, we introduce a novel approach to compare two platforms based on the calculation of differences between expression ratios observed in each platform for each individual transcript. This approach results in a concordance measure per gene (with statistical probability value), as opposed to the commonly used overall concordance measures between platforms.

**Conclusion:**

We can conclude that intra-platform correlations are generally good, but that overall agreement between the two platforms is modest. This might be due to the binomially distributed sampling variation in SAGE tag counts, SAGE annotation errors and the intensity variation between probe sets of a single gene in Affymetrix GeneChips. We cannot identify or advice which platform performs better since both have their (dis)-advantages. Therefore it is strongly recommended to perform follow-up studies of interesting genes using additional techniques. The newly introduced between-ratio difference is a filtering-independent measure for between-platform concordance. Moreover, the between-ratio difference per gene can be used to detect transcripts with similar regulation on both platforms.

## Background

Methods for the analysis of gene expression profiles have gone through progressive development over recent years. Traditionally, the level of transcribed mRNA has been analyzed using methods such as Northern blots, quantitative RT-PCR, differential display [1,2], representational difference analysis [3], total gene expression analysis [4] and suppressive subtractive hybridization [5,6]. All these methods, although fruitful and still in use, have a limited scope with regard to the number of genes that can be analyzed simultaneously. Because of this limitation, new methods have been developed, including serial analysis of gene expression (SAGE) [7], massive parallel signature sequencing (MPSS) [8], cDNA and oligo microarray chip technologies [9-13] and Affymetrix GeneChips [11].

SAGE is based on the high-throughput sequencing of concatemers of short (13–14 bp; recently 21–25 bp) sequence tags that originate from a known position within a transcript and therefore theoretically contain sufficient information to identify a transcript [7]. In contrast to microarrays, SAGE estimates the abundances (expression levels) of thousands of transcripts without prior knowledge of the transcripts being expressed. The proportion of the tag within the total number of tags in the library gives a direct estimate of the abundance of the transcript within a biological sample. The advantage of the SAGE technique is that it performs a random sampling from the pool of all expressed transcripts (also called a transcriptome) allowing the discovery of new transcripts. The proportional nature of the data enables easy exchange among researchers thus allowing the creation of large public SAGE data sets for numerous human tissues, both normal and diseased [14,15]. Disadvantages of SAGE are that the technique is expensive, labor-intensive and prone to sequencing errors. Moreover, the annotation of the short 10 bp sequence tags may identify more than one transcript. This can be overcome by using LongSAGE libraries that contain 17 bp tags which can be more reliably mapped to Unigene clusters or the complete genome sequence [16]. However, SAGE is not suitable for high-throughput analyses of multiple samples.

In contrast to SAGE, DNA microarrays are used to measure relative expression levels between samples of thousands of known transcripts. Currently, three array variants are being used (for reviews see [17,18]) i.e. spotted cDNA microarrays, spotted oligonucleotide microarrays and synthesized oligonucleotide microarrays (Affymetrix GeneChips). The advantages of Affymetrix GeneChips are that they are highly sensitive enabling the detection of mRNAs present at levels as low as 1 transcript in 100000 [11] when the probe labeling step is not considered [19]. They are suitable for high-throughput analyses of multiple samples, and data can easily be shared and used for comparisons with other researchers using the same chips. Disadvantages of Affymetrix GeneChips are that they are only commercially available, are costly and require expensive specialized equipment and are inflexible in design (although custom design is possible at high cost). Furthermore, GeneChips only measure the expression of genes represented on the chip in contrast to SAGE, in which the expression profile of the complete transcriptome can be mapped.

**Figure 1:**
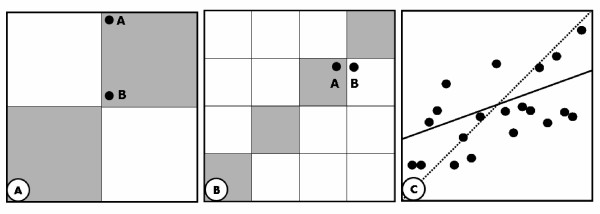
**Illustration of the methods used for the comparison of expression profiles from different platforms**. A: Up/Down classification: The points A and B with very different ratios are both considered to reflect a common tendency; B: contingency table diagonal: The points A and B, with very similar ratios, end up in different classes; C: correlation coefficients: The solid line fits to the point cloud which has a significant correlation coefficient between X and Y. However, the dashed line (Y = X) is the expected line when both platforms show identical expression patterns.

At present, SAGE, oligo microarrays, cDNA microarrays and Affymetrix GeneChips are the most widely used techniques for determining gene expression levels and gene expression ratios in different disease states and in cells under different physiological conditions or environmental stimuli. Often these different gene-expression profiling platforms are being used in parallel and data generated with the different techniques need to be compared, and possibly interchanged, within and between laboratories. Due to the overall difference in platform design, transcript level estimation, and gene annotation, direct comparisons are difficult and only a few attempts have been made to compare these different platforms (Figure 6). To determine the overall correspondence between expression levels or expression ratios of two different platforms several methods have been used in literature (Figure 1A,B and 1C). These include the parametric (Pearson) or non-parametric (Spearman) correlation coefficients between platforms, and contingency tables with varying numbers of classes for each platform. For the latter a correspondence measure can be calculated as the percentage of transcripts falling in the cells on the diagonal (Figure 1B). An extreme form of the contingency table has only 2 classes per platform (ratios above and ratios below 1) and therefore only 4 cells. This form of concordance estimation is dubbed "Up/Down classification" (Figure 1A). None of these correspondence measures was deemed satisfactory because they either treat very different ratios as similar (points A and B in figure 1A). This, in our view, makes the Up/Down classification very unreliable as an agreement measure. The use of contingency tables with more classes is already a better approach, but still some genes will be considered to be "in disagreement" while they have nearly corresponding expression ratios (points A and B in figure 1B). The Pearson correlation coefficient is a measure for the fraction of variation in Y that is explained by the variation in X, and as such, only gives a measure for the tendency of the plotted points to increase simultaneously (solid line, Figure 1C). Because of the large number of points, a slight linear regression of Y on X will give a highly significant correlation coefficient. However, when studying the correspondence between gene expression platforms, the expected linear relation has a slope of 1, when the results of both platforms are in complete correspondence (dashed line, Figure 1C), and the deviation of the observed scatter plot from this expected relation should be tested. Neither the linear Pearson, nor the Spearman rank correlation coefficient is suited for such a test. Although the fit of the point cloud to the Y = X relation can be easily calculated, the resulting statistic would still only provide a goodness of fit measure for the whole data set without giving any information on the correspondence per gene. To remedy these pitfalls we will introduce a correspondence measure based on the difference between the log(ratio) values in the two platforms for each individual transcript. Apart from serving as the basis for a measure for overall platform concordance, this method also provides the user with an agreement measure for each individual transcript which is of more interest than the overall correlation.

In the current study we have determined the similarity between SAGE- and Affymetrix GeneChips-generated gene expression profiles of two independent RNA samples. One RNA sample is isolated from a Wilms' tumor; the other is the Stratagene Universal reference RNA. These expression data were then used to evaluate the annotation problems when comparing different gene profiling platforms and the methods that can be used to compare two different platforms with respect to individual gene expression measurements and with respect to between-sample gene expression ratios. Finally, it is demonstrated that the between-ratio difference can be applied to select those transcripts that display similar expression changes in both platforms.

## Results

### SAGE data analysis

**Figure 2:**
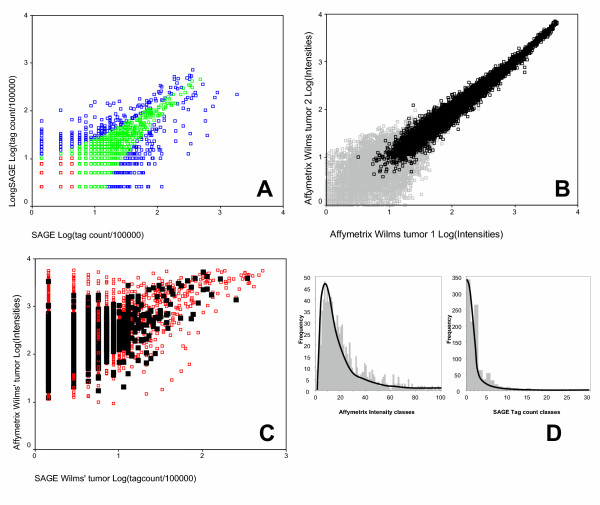
**Evaluation of gene expression in Wilms' tumor tissue. **The comparison of SAGE and Affymetrix duplicate samples demonstrates the reproducibility in both platforms (A, B). In addition, gene expression was compared between platforms (C) and showed a wide range of variation. The frequency distributions of gene expression values the final data sets do not differ from the total distributions (D). A: Comparison of a SAGE versus a LongSAGE library of the same sample Blue dots represent tag counts that are significantly different between the two libraries (according to the Z-test, Kal et al. 1999), green and red dots represent tag counts that do not differ between libraries. The red spots are tag counts that do not significantly differ from tag count 0 within the specified library (See also Table 4). B: Comparison of a duplicate analysis of one Wilms tumor sample using Affymetrix HG-U133A GeneChips. Gray spots represent probe sets that have an absent call. C: Comparison between SAGE and Affymetrix GeneChips for the Wilms' tumor sample. Red spots represent the total matching data set (n = 6408) and black spot represent the final selection (n = 1094). D: Frequency distribution of the Affymetrix intensity and SAGE tag counts from the final matched data set (1094 Unigene clusters) and the total matching data set. The smoothed line represents the distributions of the total data set in each platform. For both Affymetrix (classes with an intensity width of 10) and SAGE (classes based on tag counts) the distributions of the final data set and the total data set do not differ from each other (Chi-square values of 327 (df = 323; P = 0.412) and 104 (df = 105; P = 0.506), respectively).

In order to compare SAGE with other gene expression profiling techniques we created a SAGE library with 69792 tags from a Wilms' tumor sample. SAGE data (51954 tags) for the Stratagene Universal reference RNA (GSM1734;[20]) were obtained from the NCBI website. All tag counts are after removal of duplicate dimers and linker sequences. Within the SAGE libraries we could identify 25052 and 17497 unique SAGE 10 bp tags, for the Wilms tumor sample and the Stratagene sample, respectively. Tags can be divided into tags with low abundance (1–5 tags per 100000), intermediate abundance (6–50 tags per 100000), and high abundance (more than 50 tags per 100000). In each of the libraries, these categories contained on average 84%, 15% and 1% of the total number of unique tags (Data not shown). In addition, we created a LongSAGE library of the Wilms tumor sample for annotation purposes (as described below) and not for the comparison with Affymetrix GeneChips. This library could be used as a technical replicate of the 'short' SAGE library. Comparison of the SAGE and LongSAGE libraries showed a Pearson Correlation coefficient of 0.651 (P < 0.01) and using Z-test statistics [21] the two libraries only differed significantly from each other in 3% (α = 0.05) or 0.6% (α = 0.001) of the tags (Figure 2A). The observed differences in the LongSAGE library versus the normal SAGE library might be due to treatment with different linkers, tagging enzyme (*Mme*I instead of *Bsm*F1) and elimination of a blunt-end ligation. The pattern of variation in figure 2A closely resembles the variation predicted by the binomial distribution [22] of SAGE tag counts with only the 3% significantly different tag counts (blue dots; α = 0.05), falling outside the range of critical values. Overall SAGE and LongSAGE give identical results.

### Microarray analyses

Microarray experiments were performed using Wilms' tumor RNA and the Stratagene Reference RNA. Results of biological replicas of each sample, with independent cRNA synthesis and hybridizations, showed a good reproducibility (Pearson correlation coefficients of 0.982 (n = 11938) and 0.979 (n = 10489);both P < 0.01) using intensity values for all probe sets with a "present" signal (on average 54%; absent = 44% and marginal = 2%) (Figure 2B; black spots). This indicates that two identical RNA samples perform very similar within the pre-processing and final hybridization reactions. Although, in contrast to SAGE, the intensity signals on the array do not represent the actual abundance of mRNA molecules, we classified the Affymetrix data to get an impression of the signal distribution. These distributions are similar to those of the SAGE data. The majority (~90%) of the probe sets showed low signal intensity.

### Annotation problems

In the comparison of data obtained by SAGE and Affymetrix GeneChips only reliably annotated tags can be included (as described in the 'Matching of platforms' paragraph of the Material and Methods section; see also Shippy et al.[23]). Annotation of SAGE tags to genes and their corresponding Unigene cluster numbers revealed that on average 30% of all tags (including low abundant tags) could be reliably annotated based on the SAGE Genie principles [24]. Annotation improves to an average of 70% for tags that have an intermediate to abundant expression level. The remainder of the tags could not reliably be associated with a gene or Unigene cluster because they were not available through the SAGE Genie site, annotated to unclustered ESTs, or their reliability was below 67% (according to the SAGE Genie principles). Additionally, we performed LongSAGE for the Wilms' tumor sample, which allows the identification of 17 bp tags instead of 10 bp tags. Theoretically, over 99.8% of the 17 bp tags are expected to occur only once in the human genome. However, analyses based on actual sequences have demonstrated that only 75% of the 17 bp tags occur only once in the human genome, with the remaining tags matching duplicated genes or repeated sequences [16]. Complete annotation of LongSAGE tags using SAGE Genie data and principles revealed that 28% of all tags could be assigned a reliable Unigene cluster. Similar to SAGE, the annotation improves to approximately 70% for tags that have an intermediate to abundant expression level.

**Figure 3:**
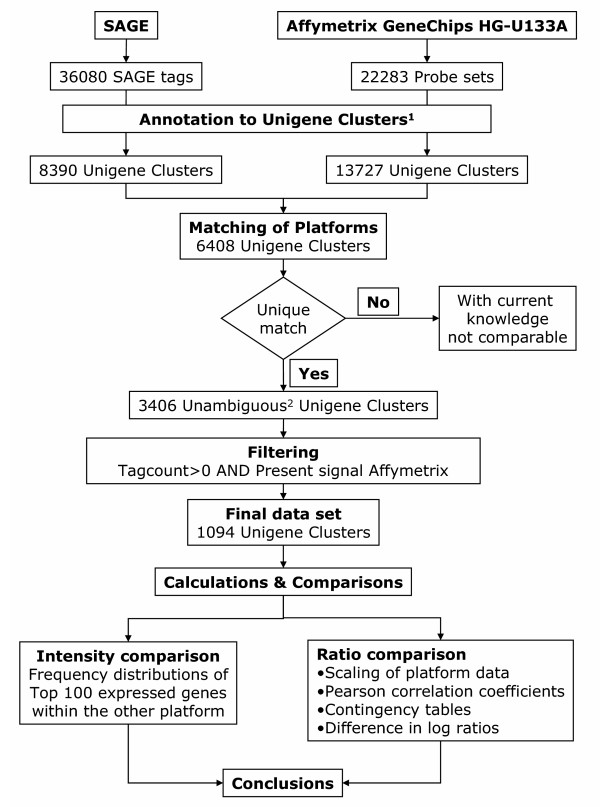
**Flow chart for matching data from two gene expression platforms. **SAGE tags were converted into Unigene clusters using data from the CGAP website. Accession numbers from Affymetrix GeneChips were also converted to their corresponding Unigene cluster. Platforms are matched according to their Unigene cluster and only unambiguous Unigene clusters are selected. Finally, data are filtered for tag counts >0 and present calls on microarray platforms. ^1. ^In the complete process of annotation a large number of tags or probe sets lost due to the following reasons: SAGE: 11733 tags with no annotation, 13113 tags with no reliable annotation, 913 tags with multiple Unigene Clusters, 80 tags belonging to linker sequences, 20 tags belonging to repetitive sequences, 22 tags belonging to mitochondrial DNA; Affymetrix: 1795 Probe sets no longer belong to a Unigene Cluster (Build 160). The remaining 20488 probe sets represent 13727 unique Unigene clusters. ^2. ^Unambiguous Unigene clusters refer to those clusters that occur only once within each platform.

The Affymetrix HG-U133A GeneChips contained probe sets for 13727 Unigene clusters that could be identified, whereas eight percent of the probe sets (i.e. 1795 probe sets) could not be linked to a Unigene cluster because these sequences are withdrawn or because these sequences are currently under revision. Figure 3 gives a schematic representation of the matching of SAGE and Affymetrix HG-U133A GeneChips data with additional information about the number of Unigene clusters within each platform, number of unambiguous Unigene clusters in each comparison and the Unigene clusters included in the final comparison. This final comparison contains 13% of the SAGE Unigene clusters and 8% of the Affymetrix Unigene clusters. These data represent 32% of the unambiguous Unigene clusters. Because of the above-mentioned problems and restrictions, only 1094 tags and probe sets were uniquely matched to the same Unigene clusters and were 'present' in both tissue samples and platforms. This relatively low number underscores the major problem in "how to merge different expression platforms". However, in view of the following quantitative comparison of gene expression platforms it is important to note that a comparison of frequency distributions of all clusters and of the selected clusters showed that the final selection of 1094 Unigene clusters does not represent a biased sample neither for the SAGE tag counts, nor for the Affymetrix array intensities. This is illustrated in figure 2D in which the frequency distributions are given for Affymetrix intensities and SAGE tag counts from the final data set of 1094 Unigene clusters. The smoothed line, which represents the frequency distribution of all SAGE tag counts and all Affymetrix intensity data (only present calls), does not differ from the distribution of the subset included in the comparison of the two platforms.

### Comparison of gene expression levels

In the comparison of platforms, we first analyzed the similarity of gene expression levels between SAGE and Affymetrix data in one tissue sample. Both datasets were matched according to their Unigene cluster numbers. Figure 2C shows a scatter plot of SAGE and Affymetrix gene expression values of the 6408 Unigene clusters before exclusion of ambiguous matches (red spots). For multiple matches, the highest tag count or intensity value per cluster was plotted. In this scatter plot the black spots represent the final selection of 1094 unambiguous and filtered Unigene clusters. Note that high Affymetrix expression levels are observed for low SAGE tag counts (spots in top-left quadrant of figure 2C), but that no high tag counts are found for low Affymetrix data (few spots in bottom-right quadrant). Overall, the correlation between SAGE tag counts and Affymetrix intensity levels of the 6408 matching Unigene clusters seemed to be modest. This was confirmed by mapping the distribution of the top 100 highly expressed genes in SAGE in the distribution of the Affymetrix dataset, and *vice versa *(Data not shown, but this can be inferred from figure 2C). In both comparisons, only halve of the genes from the top 100 of one platform have a rank in the top 100 of the other platform, whereas approximately 10% are matched to genes with ranks of over 1000 in the other platform. This already shows that the correlation of expression levels between platforms is modest.

### Comparison of between-sample expression ratios

In most gene expression studies, alterations of expression levels are expressed in relation to the simultaneously determined expression level of a reference sample and conclusions are drawn based on these ratios. To this end, expression ratios were calculated between the reference RNA and the Wilms' tumor data for the SAGE tag counts as well as for Affymetrix HG-U133A GeneChips spot intensities. In this comparison the final data set containing only the between-sample ratios for unambiguous transcripts was used (Figure 3), allowing effective comparison of the two platforms.

**Figure 4:**
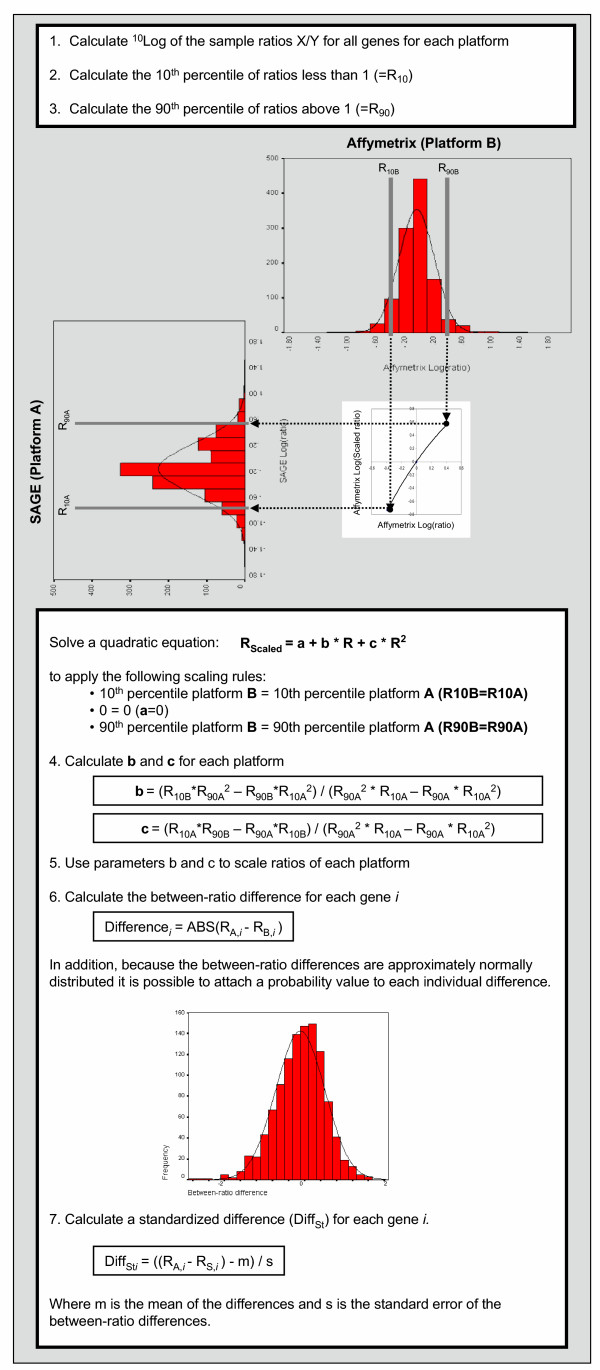
**Scaling of two gene expression profiling platforms. **Illustration of the steps involved in the scaling of values in each of the platforms to a common scale. The procedure takes the ratio distribution in one of the platforms and scales the other to match the same range of ratio values using a quadratic equation based on ratio 1 and the 10^th ^and 90^th ^percentile values of each platform. The (scaled) ratio values are then used to calculate between-platform ratio differences per transcript. In addition, it is demonstrated how the ratio differences can be used to calculate the standardized between-platform log(ratio) difference and a probability value. For further details: see the Materials and Methods section.

**Figure 5:**
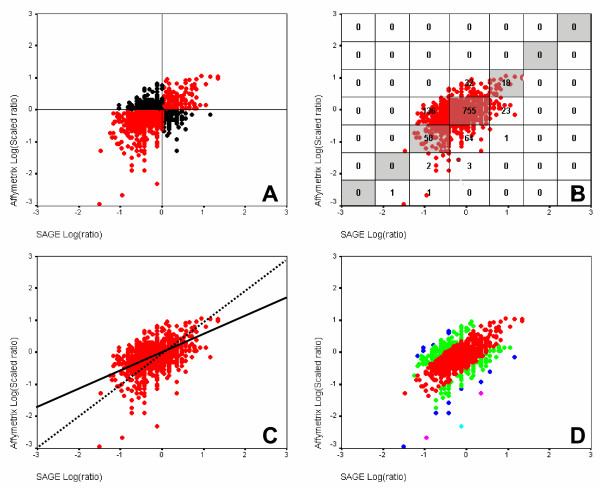
**Comparison of SAGE and Affymetrix HG-U133A GeneChips results using the scaled ratios between Wilms' tumor and Stratagene Universal Reference RNA expression levels.** A: Up/Down classification. The red points in the upper-right and lower-left were considered to be in agreement between the platforms. B: contingency table diagonal based on the classification of gene expression ratios into log (10-fold) classes. The genes falling in the classes on the diagonal were considered to be in agreement between the platforms. C: Pearson correlation coefficient. The correlation coefficient was 0.472 and corresponds to a linear regression line with a slope of 0.492 (solid line) The Y = X line with a slope of 1 (dashed line) is the expected line when both platforms have identical expression patterns. D: absolute between-platform ratio differences (see Figure 4) were calculated and classified: 0–0.5 (red), 0.5–1.0 (green), 1.0–1.5 (blue), 1.5–2.0 (magenta), 2.0–2.5 (light blue). These classes represent an approximate less then 3, 10, 30, 100, and 300-fold difference, respectively, between the two platforms. The points in the 0.5 zone were considered to be in agreement between the platforms.

To enable direct comparisons of ratio measurements using different gene expression platforms, the ratios of the Affymetrix platform were scaled to those of the SAGE platform as described in Figure 4 ("scaling of two platforms"). In addition, different approaches were used to describe the correlation of the resulting scaled gene expression ratios between platforms (Figure 5). For the comparison of gene expression ratios based on contingency tables we used two approaches, i.e. Up/Down classification (Figure 5A) and a contingency table diagonal based on intensity classes (Figure 5B). These comparisons lead to an agreement of 63% and 76% between platforms, respectively. Furthermore, the Pearson correlation coefficient, calculated as a measure for the agreement between platforms, was 0.453 (P < 0.01). Regression analysis shows a linear trend with a slope of 0.477 for Affymetrix versus SAGE, which according to the correlation coefficient differs significantly from a slope of 0. However, this slope also deviates significantly from the slope value of 1 which is expected when the platforms are identical (t-test for slopes; P <0.001; Figure 5C). Finally, we compared SAGE and Affymetrix data using our proposed classification based on the difference between the two ratios per Unigene cluster. When we accept a 0 to 3-fold difference as indicative for agreement between the two platforms (red points in figure 5D), this approach showed that the two platforms have an agreement of 78%.

**Figure 6:**
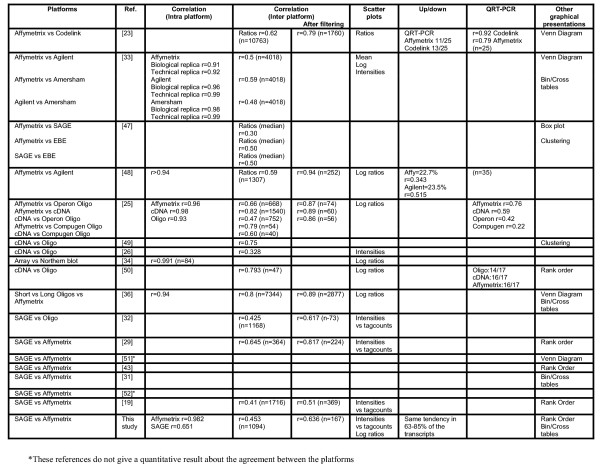
Literature overview of platform comparisons

Like others have demonstrated (Figure 6) the overall agreement between platforms improves when only highly expressed transcripts (based on their tag counts) are included (Table 1). When only lowly expressed genes were included the concordance based on the contingency table diagonal and correlation coefficient steeply decreased whereas the other measures were both hardly affected. Inclusion of only those tags that were significantly differentially expressed between the two samples markedly improved the Up/Down classification and correlation coefficient based measures. Note that the concordance measure based on the between-ratio difference was least affected by these selections. This indicates that this new measure is robust and less dependent on filtering than the other overall measures.

**Table 1:** Summary of similarities between SAGE and Affymetrix HG-U133GeneChips for the final dataset (= 1094)

	**UP/DOWN classification**	**Contingency table diagonal**	**Pearson Correlation coefficient^3^**	**0–3 fold between-ratio difference**	**N**
**All transcripts**	63%	76%	0.453	78%	1094
**Low expresssion^1^**	57%	81%	0.222	78%	572
**High expresssion^1^**	69%	81%	0.578	90%	226
**Significant difference^2^**	86%	47%	0.636	70%	167

^1. ^Based on the binomial sampling error of SAGE tags, tags counts below 5.7 and 7.7 (per 100,000) for the WT and Stratagene sample, respectively, are not significantly different from tag count 0. When a tag falls below these thresholds in both libraries it is included in the "Low expression" group (line 2); when a tag counts is above these thresholds in both libraries it is included in the "High expression" group (line 3). The thresholds were calculated as the 95% confidence interval of the tag proportion: *CI*_95%_) = *n *± 1.96*  with n = tag count; N = Library size and p = n/N (proportion)

^2.^Significant difference between the two SAGE libraries is defined as a significant P-value (α<0.05) according to the Z-test between two libraries [21].

^3.^All observed correlation coefficients are significant at P < 0.01

### Sources of differences in gene expression ratios

In an attempt to explain the difference in gene expression between SAGE and Affymetrix GeneChips we summarize different sources. Variation due to "noisy fold ratios" generated from low-intensity transcripts is a widespread cause of error when computing statistics on ratios without accounting for the intensities from which the ratios were derived [25]. Within our data set we have shown that the final data set is an unbiased selection of the total data set (Figure 2D). Additionally, the mean intensity signals for both SAGE and Affymetrix GeneChips appear to be randomly distributed over the ratio distribution (data not shown). This indicates that the difference in expression ratios between platforms is not caused by low intensity values.

In addition, it has been suggested that the GC-content of the transcripts could influence the correspondence between platforms [26]. To test this hypothesis for the final data set (n = 1094) we retrieved all transcript sequences (mostly Refseq sequences [27]) and probe set sequences and calculated the GC-content for each transcript and the average GC-content of the corresponding probe sets. The GC-contents were divided into classes (30–35%; 35–40%; 45–50% etc.) and the correlation between GC-content and the differences in expression ratios between platforms was tested. Statistical analysis showed that ratio differences did not depend on the GC-content of the transcript (Chi-square value of 25.69; df = 35; P = 0.875). However, Unigene clusters showing good agreement between platforms tend to depend on the high GC-content of the corresponding probe sets (Chi-square value of 61.114; df = 30; P = 0.001). This GC-analysis indicates that expression data from probe sets with a higher GCcontent show a better agreement with their corresponding SAGE data and are more reliable. Note in this respect that for a Unigene cluster the GC content of a probe set is not necessarily the same as that of a transcript.

## Discussion

To answer the question whether gene expression data generated by SAGE and by Affymetrix HG-U133A GeneChips can be used interchangeably, data from these two techniques were compared using two independent RNA samples. Analysis of intra-platform variation shows good correlation for both SAGE and Affymetrix; this is also observed by others (see Figure 6). The inter-platform comparison depends on reliable annotation of the SAGE tags for which we used the tag annotation from SAGE Genie [24]. A reliable association could be made only for 30% of all tags, which increases to 70% for intermediate and high abundant tags. This indicated that SAGE tag annotation requires improvement, especially for low abundant tags. Based on literature findings, the use of LongSAGE should refine annotation of SAGE tags [16]. However, the current study showed an annotation profile similar to the above-mentioned percentages, indicating that LongSAGE is still not sufficient for unique gene identification. Similar disappointing improvements in annotation efficiency have been found in other studies [19]. Further comparison of SAGE and LongSAGE requires a study that falls beyond the scope of this paper; such a study has recently been published [19]. For the annotation in Affymetrix GeneChips, accession numbers had to be converted to Unigene clusters, which was hampered by the fact that 8% of the transcripts present on Affymetrix HG-U133A GeneChips were no longer present in a Unigene cluster. Moreover, some probe sets might represent a different transcript than initially reported (see for an example [28]).

A first impression about the agreement between SAGE and Affymetrix HG-U133A GeneChips was obtained from the evaluation of the top100 of highly abundant transcripts in one RNA sample in each platform. This comparison showed that approximately 50% of the top100 of highly expressed transcripts showed a corresponding expression within the top100 of highly expressed transcripts of the other platform. This is in line with the findings of Ishii et al. [29] who compared SAGE with Affymetrix GeneChips containing approximately 6000 transcripts, and Iacobuzio-Donahue et al. [30] who showed that only genes that display robust changes in gene expression were identified by both platforms. In our current study, approximately 80% of transcripts detected in the top100 of one platform were mapped within the top1000 of the competing platform. A similar figure was presented by Evans et al. [31] who used the RG-U34A Affymetrix GeneChips. Recently, Kim [32] suggested that absolute expression analyses of SAGE and oligonucleotide microarray technology reliably detected medium-to-high abundant transcripts.

For a more extensive comparison between the individual gene expression profiling platforms we used gene expression ratios between Wilms' tumor and Stratagene Universal reference RNA as determined by SAGE and Affymetrix GeneChips. The use of ratios might have the disadvantage of losing information about individual expression values. However, it corrects for platform specific variations (i.e. probe design, hybridization efficiencies etc.). By matching SAGE and Affymetrix data, an unambiguous data set was generated. On average about 30% of the unambiguous genes were observed to be expressed by both SAGE and Affymetrix GeneChips and could be included in the final comparison. Although this comparison comprised only 13% of all SAGE Unigene clusters and only 8% of the Affymetrix Unigene clusters, it was demonstrated that this selection was unbiased with respect to gene expression levels in each of the platforms. This allows the extrapolation of the conclusions to the whole platform.

We looked for the correspondence in gene expression results between the two techniques using Up/Down classification (Figure 1A), the contingency table diagonal (Figure 1B) and correlation coefficients (Figure 1C). In addition, an approach was introduced in which differences between scaled ratios were calculated. The latter measure was introduced to circumvent pitfalls of Up/Down classification, contingency tables and correlation coefficient that were discussed in the background section. To this end, we introduced an approach in which the scaling of the ratio data enables the calculation of individual ratio differences between platforms. These ratio differences can then be used to determine to which extend and in which range (e.g. 0–3 fold difference) two platforms differ in their expression ratio estimation. In this study we show that, as opposed to the other overall concordance measures, the between-ratio difference is hardly sensitive to filtering of noisy data. From the current analysis, we conclude that contingency tables and, preferably, calculation of ratio differences between two platforms should be used to compare gene expression profiles from different platforms. Moreover, the between-ratio difference provides the user with a correspondence measure per individual gene that can be used to select those genes for which a predetermined correspondence level is reached. The approximately normal distribution of the between-ratio differences (Figure 4) allows the calculation of a standardized difference value for each gene from which a P-value can be obtained. Note that this P-value cannot be used to test whether the ratio difference equals zero. Such a test requires a gene specific variance estimate in the denominator of the standardized difference and such a variance estimate cannot be obtained from the four non-replicated expression values that are used to calculate the ratio difference. However, the standardized difference and its P-value can be used as a measure for the position of a specific gene within the distribution of between-platform ratio differences and as such they can serve as a statistical threshold to determine which genes can be confidently interchanged between platforms. For instance, in the current study, the transcripts with a less than 0.5 fold between-ratio difference (red dots in figure 5D) have a chance of at least 0.8 that they show similar gene expression on both platforms. Some of the choices in the scaling procedure can be considered to be ad-hoc. However, given the current state of understanding of the causes for within and between platform variability it was deemed best to opt for a simple quadratic scaling equation to convert the distribution of ratios, which is asymmetric around 1 to a common scale. When the knowledge on the physics, chemistry, and sampling statistics increases, better conversion functions will present themselves.

The overall similarity between SAGE and Affymetrix GeneChips is modest when expression ratios are compared. The correspondence improves to 90% when only highly expressed transcripts are included which means that noise is filtered out for both platforms. The differences between SAGE and Affymetrix GeneChips were not caused by a biased selection of the final data set, differences in GC-content of the included transcripts or extreme ratios resulting from low gene expression values. The observed cross-platform differences, arise from intrinsic properties of the platforms themselves, differences in the principle of determining the expression levels, such as absolute (SAGE) versus quantitative (microarray) mRNA levels, and/or processing and analytical evaluation [33]. These disparities of the two technical approaches are summarized in table 2 and may all contribute to the modest overall correlation of SAGE and microarray data. We cannot conclude which of the platforms performs best. These results show, as also argued by Tan and co-workers [33], that it is important to validate the results obtained with SAGE or Affymetrix GeneChips with subsequent northern blots or quantitative PCR analysis [34-36]. It was beyond the scope of our analysis to perform such a verification of expression data. Anyway, such a validation is impractical for large numbers of genes. However, it seems that the divergence of the SAGE and Affymetrix platforms in this study is for a large part due to the wide range of Affymetrix gene expression values observed for transcripts with a low gene expression level in SAGE (Figure 2C). A similar over-representation of high Affymetrix expressions for low SAGE tag counts has been published by Lu et al. [19]. We currently showed that a SAGE and LongSAGE library from the same RNA sample showed nearly identical expression profiles (Figure 2A). These findings confirm the results found within direct comparisons of SAGE libraries [37-39]. In addition, the differences between SAGE and LongSAGE can be fully explained by the binomial distribution of the sampling error in individual SAGE tag counts [22]. Therefore, it can be ruled out that many low SAGE tag counts originate from high abundant transcripts. This is also confirmed by Sun et al. who demonstrate that 70% of the low-copy SAGE tags represent real low level transcripts [40]. The Affymetrix platform showed highly reproducible intensity values when applied twice to the same tissue sample. However, because of the variation between probe sets per Unigene cluster [25] it cannot beruled out that some Affymetrix probe sets provide systematically biased intensity levels and expression ratios. It is a known problem that different probe sets belonging to the same transcript show variation in expression detection. Several explanations have been given for this variation: (1) probe sets may represent splice variants or may cross-hybridize to different members that belong to a highly similar gene family or transcripts with different poly-A sites; (2) one probe set is more 5' located than the other and (3) one probe set is better designed than the other [41]. Such a bias might explain the weak correspondence between the SAGE and Affymetrix platform observed in this and other studies [19,23,25]

Future studies should be aimed on improving the efficiency of SAGE tag annotation and avoidance of systematic bias in microarray techniques. Only then, measurements of various technologies can be directly compared and transformed to a universal gene expression catalogue. SAGE has the advantage that a whole transcriptome is analyzed, but is limited to the analysis of a small number of samples. For screening of large sets of samples SAGE cannot be the favored choice and Affymetrix GeneChips might be a good alternative. Therefore, we think that the future lies in combining the data from SAGE with Affymetrix GeneChips, custom cDNA or oligo arrays. This gives the advantage of complete expression profiling using SAGE and high-throughput array screening of a larger panel of samples allowing rapid identification and for instance validation of clinical relevant genes involved in disease onset [42,43]. Finally, the proposed ratio difference between platforms using an universal reference sample (as also indicated in [25]) can serve as a measure for interplatform correspondence per individual gene.

**Table 2:** Disparities of the technical approaches

**SAGE**

• Sequence errors (although it has been shown that most of the single-copy SAGE tags are not generated from experimental sequence errors, but that they are novel tags derived from novel transcripts [53])
• Tag annotation difficulties
• Missing transcripts due to absence of a recognition site for the anchoring enzyme (approximately 0.7%) or GC-content bias [24,54]
• Incorrect tags arise from incomplete digestion or alternative poly-adenylation [55]
• Sequence polymorphisms resulting in multiple tags for a single transcript

**Affymetrix HG-U133 GeneChips**

• Probe design issues (such as distance of the target sequence from the poly-A tail; secondary structures within the target sequence; cross-reactivity of the probe with other transcripts, nucleic acid structure)
• Differences in hybridization efficiencies between probe sets
• Incorrect annotation of transcripts (no sequence verification)
• Efficiencies in dye incorporation

## Conclusion

This paper evaluates several approaches for the comparison of different gene expression platforms, outlined using SAGE and Affymetrix GeneChips. We demonstrate that for both SAGE and Affymetrix GeneChips the intra-platform correlations are extremely good, but that the inter-platform agreement based on an unbiased selection of transcripts is modest. The agreement between platforms increases if only transcripts are included with high tag counts and high hybridisation intensities. It appears that the expression distributions are similar for each of the platforms, but that the correlation between platforms is modest due to intrinsic differences, like sensitivity, levels of noise, and gene annotation. Finally, we introduce a novel, filtering-independent approach for data analysis based on the calculation of differences between expression ratios observed in SAGE and Affymetrix GeneChips for each individual transcript. The statistical probability value that can be assigned to each individual betweenratio difference, allows the selection of individual transcripts that display similar regulation on both platforms.

## Methods

### Tissue and RNA extraction

Wilms' tumor tissue was obtained from a single individual after resection of the tumor. Tissue was immediately frozen in liquid nitrogen. Informed consent to use this material for scientific research was obtained. After homogenization, total RNA was extracted using Trizol (Invitrogen, Breda, The Netherlands), dissolved in RNase free water and stored at -80°C. The Stratagene Universal reference RNA was obtained from Stratagene (Stratagene, Amsterdam, The Netherlands, catalog #740000-41). Purity and integrity of the RNA samples was confirmed on the Agilent 2100 Bioanalyzer (Agilent Technologies Netherlands B.V., Amstelveen, The Netherlands), using the LabChip^® ^approach.

### Construction of SAGE libraries

The SAGE library of the Wilms' tumor RNA was generated using the I-SAGE kit according to the manufacturer's instructions (Invitrogen, Breda, The Netherlands; cat. #T5000-03). A detailed protocol may be obtained as a free download [44]. For LongSAGE minor modifications were implemented in the protocol of the I-SAGE kit; i.e. the restriction enzyme *Bsm*FI was replaced by *Mme*I, linkers were adapted for LongSAGE and ditags were created using sticky-end ligation. All sequence files were processed using the SAGE2000 software provided by Dr. K.W. Kinzler (see also [45]). The SAGE library from the Stratagene Universal reference RNA was obtained from the NCBI website. This library can be retrieved in the Gene Expression Omnibus under code GSM1734 [14,20]).

### Annotation of tags

Extracted SAGE tags were annotated based on the SAGE Genie principles [24] through several stringent filters using data from the CGAP website [15]. Several databases (i.e. HsMap.txt, HsRepetitive.txt and HsDatasets.txt) were combined to a final dataset containing all information necessary for tag annotation. Tags matching to unclustered EST's were considered to be no-matches. Tags matching to Unigene clusters retrieved from low ranked databases (<67%; according to the rules set by CGAP) were not included in our comparisons. During this process tags are matched to no, one unique, or more than one Unigene cluster (Unigene Build 160, March 2003). To further identify tags matching more than one Unigene cluster, we extracted the 11^th ^base from our original sequence files using the SAGE2000 software. This 11^th ^base can be used to match against the deposited sequences (Genbank, EMBL etc.) and in this way one may be able to exclude Unigene clusters that contain a different 11^th ^base in their sequence and thereby minimize the number of multiple matches. In the final comparison tags matching to multiple Unigene clusters were excluded. For annotation of LongSAGE tags we used the data available at the CGAP site for Unigene Build 170 (July 2004). These annotations were not available for Unigene Build 160.

### Affymetrix

Affymetrix HG-U133A GeneChips were used and the hybridizations were performed according to the manufacturer's protocols and carried out at the Micro-array Department (MAD; Institute for Life Sciences, Faculty of Science, University of Amsterdam). For analysis, the MAS 5.0 software suite was used and comparisons between duplicate Wilms' tumor hybridizations and duplicate Stratagene Universal reference RNA hybridizations were made (data were deposited into the GEO under accession GSE1158). This gives four comparisons (^2 ^Log ratios), from which the geometric mean gene expression ratio between the two samples was calculated. Probe sets on the Affymetrix chips were matched with Unigene clusters (Unigene Build 160, March 2003).

### Matching of platforms

The matching of data from two different gene expression profiling platforms (as illustrated in figure 3) poses a couple of problems. On the one hand, a SAGE tag may link to more than one Unigene cluster which results in matches with multiple different Affymetrix probe sets. On the other hand multiple tags originating from one Unigene cluster might match with one Affymetrix probe set. Examining all multiple matches for each individual transcript is extremely laborious and beyond the scope of this study. To circumvent these and other problems we included in our comparison only those clusters for which a one-to-one relation between the two platforms was found. These clusters are called unambiguous Unigene clusters. This matching step already results in a considerable reduction of data available for the comparison. In addition, data were filtered for the presence of gene expression (tag count>0 in both SAGE libraries and present signal on the arrays for both RNA samples).

### Comparison of expression ratios between samples

For each platform and each transcript that full-filled the matching criteria an expression ratio between Wilms' tumor and Stratagene Reference RNA was calculated. With these ratios the correspondence between platforms was estimated using the Pearson correlation coefficient, Up/Down classification and a contingency table (Figure 1A, 1B, 1C). Because none of these measures was deemed satisfactory as overall correspondence measure (see background section) we developed a new measure based on the difference between the log(ratio) values in the two platforms for each individual transcript (Figure 4). The chemistry, physics and statistics of the detection technique make that in each platform the observed gene expression is a non-linear transformation of the real gene expression level. For instance, saturation of the array hybridization makes that the high expression levels are truncated. However, because such artifacts affect genes in both tissues in the same way, an observed expression ratio of 1 can still be expected to be observed for genes that are not differentially expressed in the studied tissues. On the other hand, these saturation effects, as well as the relatively larger Poisson error in the detection of low intensity values will affect the ratios on both sides of the ratio distribution in an unpredictable way. Similarly, the sampling error in SAGE will affect ratios for lowly expressed genes, despite the fact that SAGE tag counts are linearly related to transcript abundance. The substitution of zero tag counts that is required for the calculation of ratios will also skew the ratios [46]. Finally, the discrete nature of tag counts, combined with the necessary normalization of tag counts to tags per 50000, will have non-linear effects on the observed ratio distribution in the SAGE platform. Therefore, the relation between the gene expression ratios observed in the SAGE and Affymetrix platform cannot be assumed to be a simple linear Y = X relation. This is already clear from the difference ranges of ratio values in each platform. To directly compare the ratios observed in both platforms at least the range of observed ratios should be similar. The nature of the relation is unknown and fully obscured by the variability in both platforms. However, because in each platform the observed ratio of 1 can be assumed to be true, the simplest function to scale the range of ratio of one platform to that of the other platform is a quadratic equation. Such a scaling function can be based on three values from each ratio distribution. These are the ratio of 1 and, to avoid undue influence of the extreme ratios, the 10^th ^and 90^th ^percentile values. The quadratic scaling takes into account that the ratio distribution is not symmetrical around ratio 1. The full scaling procedure is illustrated and detailed in Figure 4. Note that the scaling uses log(ratio) values. After scaling, the absolute difference between the log(ratios) per individual gene was calculated. The resulting differences of log(expression ratios) were classified into classes of width 0.5, which corresponds to an approximate 3-fold difference in expression ratio between platforms. These classes were used to label the genes in scatter plots of two different platforms (Figure 2D). As illustrated in Figure 4, the distribution of between-ratio differences is approximately normal. Therefore, the mean and standard deviation of this distribution can be used to calculate a standardized difference value (Diff_st_) per gene and a P-value for this standardized difference can be obtained from the normal distribution. This P-value can then serve as a measure for the position of each gene in the distribution of between-platform ratio differences. Note that this P-value should not be interpreted as a significance value for the ratio difference between platforms. Such a test requires a gene specific variance estimate in the denominator of the standardized difference, which cannot easily be derived from the available data.

## Authors' contributions

MK, FB and FVR planned and designed the study. JMR and FVR analyzed the data, generated the figures and drafted the manuscript. MK and FB helped by editing the manuscript, providing overall technical guidance and coordination. LA, DAZ and FVR created the LongSAGE and SAGE libraries, and performed cloning and sequencing of the concatemers. JMR developed the new approach for the comparison of multiple platforms, performed calculations with FVR and provided guidance with the statistical analyses. GJS and FVR performed the annotation of SAGE tags. All authors read and approved the final manuscript.

## Grants

This work was supported by the Stichting Kindergeneeskundig Kankeronderzoek (SKK) and the Dutch Cancer Society (KWF; grant UVA 2001–2558)
